# Patient with papillomatous lesions

**DOI:** 10.1016/j.jdcr.2025.02.004

**Published:** 2025-03-05

**Authors:** Jay Patel, Mariana Chrispim Gimenez, Mary Gail Mercurio

**Affiliations:** Department of Dermatology, University of Rochester Medical Center, Rochester, New York

**Keywords:** ectodermal dysplasia, focal dermal hypoplasia, Goltz syndrome, verrucous carcinoma

## History

**Fig 1:**
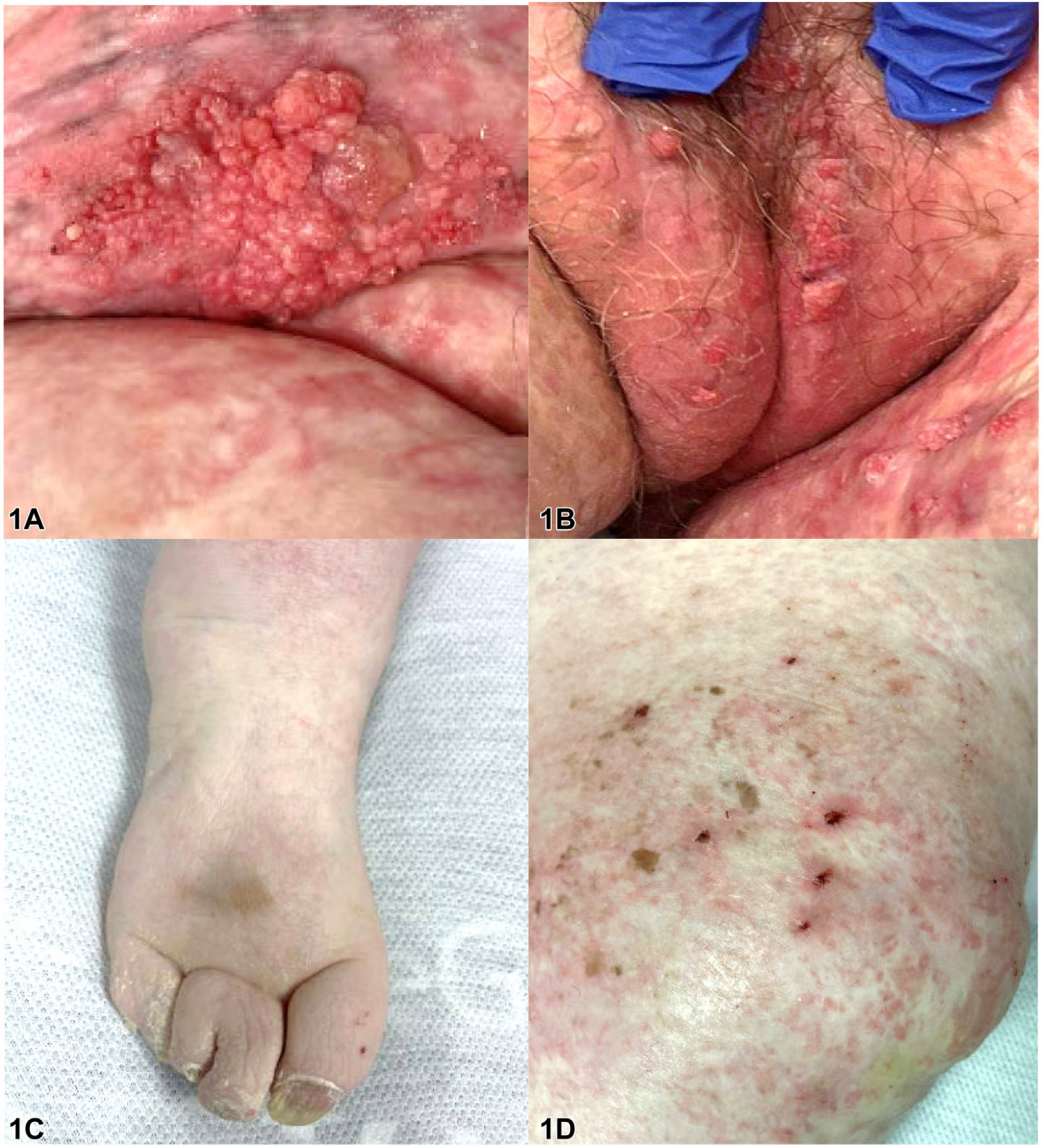

**Fig 2:**
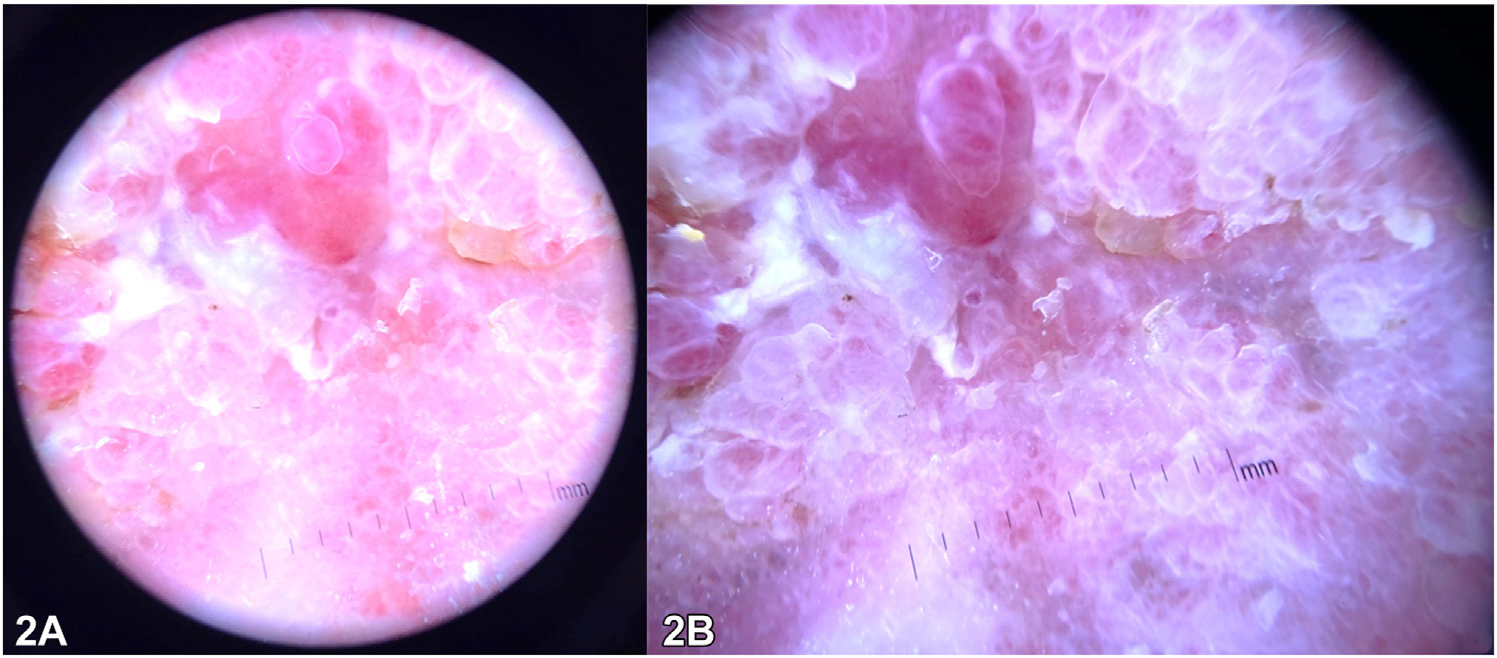

A 75-year-old woman with a history of basal cell carcinoma presented with a 5-year history of asymptomatic fleshy pink papillomatous plaques with focal overlying red-yellow hemorrhagic crust in the groin and vulva (Fig 1, *A* and *B*). Further exam showed hyperkeratotic atrophic papules to plaques on bilateral medial thighs, scattered atrophic scars, syndactyly of right toes, and longitudinal ridging of bilateral fingernails and toenails (Fig 1, *C* and *D*). Dermoscopy revealed a homogenous pin-white polylobular plaque without dotted vessels (Fig 2, *A* and *B*). Biopsy of the left medial thigh identified epidermal acanthosis, papillomatous architecture, and a central fibrovascular core.

**Question 1: What is the most likely diagnosis, given the constellation of findings described above?**

A. Condyloma acuminatum
B. Lymphangioma circumscriptum
C. Epidermal nevus
D. Vascular papilloma
E. Verrucous carcinoma

**Answers:**

A. Condyloma acuminatum – Incorrect. While genital warts can appear similar in presentation and location, the constellation of physical exam findings favors a diagnosis of Goltz syndrome. Vascular papillomas are frequently seen in these patients and may resemble condyloma. However, the dermoscopic presentation of the condyloma acuminata often contains more vascular elements, such as dotted or glomerular vessels, which are not seen in the images.^1^
B. Lymphangioma circumscriptum – Incorrect. This condition is often associated with lymphatic defects and classically presents as thin-walled, translucent vesicles, filled with clear lymphatic fluid and a variable amount of blood.
C. Epidermal nevus – Incorrect. Epidermal nevi most often present in younger patients and have skin-colored to brown coloration.
D. Vascular papilloma – Correct. This patient has Goltz syndrome, also known as focal dermal hypoplasia, dermatologically characterized by dermal atrophy, vascular papillomas, pigmentary alterations, dystrophic nails (eg, longitudinal ridging, hypoplasia), and patchy alopecia of the scalp.^2^ The vascular papillomas seen in this condition present with a “raspberry” appearance, characterized by a verrucous surface and fibrovascular core, and favor the lips and anogenital area. Additional features include ocular defects (coloboma, strabismus, microphthalmia), dental anomalies (hypodontia, oligodontia), and digital abnormalities (syndactyly, polydactyly).^2^
E. Verrucous carcinoma – Incorrect. Verrucous carcinomas are characteristically more heterogenous in appearance clinically with areas of crust, necrosis, and hemorrhage. Although verrucous carcinoma can closely resemble vascular papillomas seen in Goltz syndrome and is often distinguished through biopsy, it would not explain the accompanying distinct clinical features.

**Question 2: How can the papillomatous lesions seen in this condition be managed?**

A. Ongoing monitoring
B. Cryotherapy
C. Surgical intervention
D. Laser therapy
E. All the above

**Answers:**

A. Ongoing monitoring – Incorrect. Although this is a reasonable treatment option, it is not the only correct answer. These papillomatous lesions are benign and do not require excision unless symptomatic or functionally impairing.
B. Cryotherapy – Incorrect. While cryotherapy is considered a possible treatment for these lesions, it is not the only intervention used. Furthermore, while this is less invasive than surgical excision, concerns regarding possible recurrence and feasibility for larger lesions do exist.^3^
C. Surgical intervention – Incorrect. Surgical intervention such as excision or curettage has demonstrated good results in the management of Goltz syndrome associated papillomas and is a treatment option, but depending on their location and patient preference, it might not be necessary or possible.^3^
D. Laser therapy – Incorrect. Different laser therapies, such as CO_2_ laser vaporization and pulsed dye laser, have been shown efficacious in the management of these papillomas.^4^
E. All the above – Correct. All the above treatment strategies can be used depending on patient preference, location, and functional impairment.

**Question 3: Which of the following statements regarding the inheritance pattern of this condition is correct?**

A. This disorder most often occurs due to *de* novo mutations in the *PORCN* gene, but it can also be inherited in an X-linked dominant manner, with phenotypic variability and severity associated with the degree of lyonization
B. This disorder is inherited in an X-linked recessive manner and is antenatally lethal in males
C. This disorder solely occurs due to *de novo* mutations in the *PORCN* gene
D. This disorder solely occurs due to X-linked dominant inheritance, with the severity of the phenotype being associated with the level of X-chromosome inactivation
E. The inheritance pattern for this condition has not been elucidated yet

**Answers:**

A. This disorder most often occurs due to *de* novo mutations in the *PORCN* gene, but it can also be inherited in an X-linked dominant manner, with phenotypic variability and severity associated with the degree of lyonization – Correct. This genodermatosis is caused by a mutated *PORCN* gene, which encodes porcupine o-acyltransferase and results in abnormal ectodermal tissue development. This usually develops as a *de* novo pathogenic variant (95%), but can also be inherited in an X-linked dominant pattern (5%) and present in a mosaic distribution. It also causes skewing of X-chromosome inactivation, with the degree to which it happens correlated with the phenotype.^2^^,^^5^
B. This disorder is inherited in an X-linked recessive manner and is antenatally lethal in males – Incorrect. The inheritance pattern for Goltz syndrome is discussed above. Furthermore, there are cases of live-born affected males that are mosaic for a *de novo* postzygotic *PORCN* pathogenic variant or have functional mosaicism.^5^
C. This disorder solely occurs due to *de novo* mutations in the *PORCN* gene – Incorrect. While most cases of Goltz syndrome occur due to a *de novo* pathogenic variant, a minority of them is genetically inherited.^5^
D. This disorder solely occurs due to X-linked dominant inheritance, with the severity of the phenotype being associated with the level of X-chromosome inactivation – Incorrect. While this is the correct inheritance pattern for Goltz syndrome, most cases occur due to a *de novo* pathogenic variant.^5^
E. The inheritance pattern for this condition has not been elucidated yet – Incorrect. Inheritance pattern discussed above.

## Conflicts of interest

None disclosed.
